# Genome-Wide Identification, Phylogenetic and Expression Analyses of the Ubiquitin-Conjugating Enzyme Gene Family in Maize

**DOI:** 10.1371/journal.pone.0143488

**Published:** 2015-11-25

**Authors:** Dengwei Jue, Xuelian Sang, Shengqiao Lu, Chen Dong, Qiufang Zhao, Hongliang Chen, Liqiang Jia

**Affiliations:** 1 Key Laboratory of Tropical Fruit Biology (Ministry of Agriculture), South Subtropical Crops Research Institute, Chinese Academy of Tropical Agricultural Sciences, Zhanjiang, 524091, China; 2 Maize Research Institute, Guangxi Academy of Agricultural Sciences, Nanning, 530227, China; Universidade Federal do Rio Grande do Sul, BRAZIL

## Abstract

**Background:**

Ubiquitination is a post-translation modification where ubiquitin is attached to a substrate. Ubiquitin-conjugating enzymes (E2s) play a major role in the ubiquitin transfer pathway, as well as a variety of functions in plant biological processes. To date, no genome-wide characterization of this gene family has been conducted in maize (*Zea mays*).

**Methodology/Principal Findings:**

In the present study, a total of 75 putative *ZmUBC* genes have been identified and located in the maize genome. Phylogenetic analysis revealed that ZmUBC proteins could be divided into 15 subfamilies, which include 13 ubiquitin-conjugating enzymes (ZmE2s) and two independent ubiquitin-conjugating enzyme variant (UEV) groups. The predicted *ZmUBC* genes were distributed across 10 chromosomes at different densities. In addition, analysis of exon-intron junctions and sequence motifs in each candidate gene has revealed high levels of conservation within and between phylogenetic groups. Tissue expression analysis indicated that most *ZmUBC* genes were expressed in at least one of the tissues, indicating that these are involved in various physiological and developmental processes in maize. Moreover, expression profile analyses of *ZmUBC* genes under different stress treatments (4°C, 20% PEG6000, and 200 mM NaCl) and various expression patterns indicated that these may play crucial roles in the response of plants to stress.

**Conclusions:**

Genome-wide identification, chromosome organization, gene structure, evolutionary and expression analyses of *ZmUBC* genes have facilitated in the characterization of this gene family, as well as determined its potential involvement in growth, development, and stress responses. This study provides valuable information for better understanding the classification and putative functions of the UBC-encoding genes of maize.

## Introduction

Protein ubiquitination is mediated by the sequential action of an E1 activating enzyme, an E2 conjugating enzyme, and a range of E3 proteins, which are thought to confer substrate specificity [1]. Ubiquitin-conjugating enzymes (E2s) comprise a class of eukaryotic enzymes that function at an intermediate step in the reaction pathway, leading to protein ubiquitination. E2 accepts thioester-linked ubiquitin from E1 to their own active-site cysteine via a transthiolation reaction. E2 then transfers ubiquitin either to a substrate directly aided by an E3 or to a cysteine of an ubiquitin protein ligase (E3s) through a second transthiolation reaction that then transfers ubiquitin to the substrate. E2s for ubiquitin and most UBLs contain a conserved catalytic domain of approximately 140 to 200 amino acids, called the UBC domain [2]. The required conserved cysteinyl residue for thioester formation is contained within this region. This conservation is observed in both sequence and structural comparisons of E2s. For example, the 11 E2s and two ubiquitin-like conjugating enzymes (UBC9 and UBC12) of *Saccharomyces cerevisiae* exhibit 20%–92% identity [3]. The UBC domains of different E2s have a high degree of sequence homology and adopt similar structures comprised of four α-helices, an anti-parallel β-sheet formed by four strands, and a short 3_10_–helix [4–6]. The highly conserved active-site cysteine (Cys) is located in a shallow groove formed by a short loop connecting α-helix 2 with α-helix 3 and a long loop proximal to the active site [7]. It is thought that the requirement of E2s to interact with E1 and ubiquitin (or its cognates) during E2-ubiquitin thiol ester formation places considerable evolutionary constraints on the E2 structure, resulting in the observed conservation [8].

The protein family has expanded during evolution. Lower eukaryotes have lower numbers of E2 enzymes than higher ones. All eukaryotes possess several E2s, ranging from no more than 20 in algae to a little over 40 in certain multicellular plants and animals. For example, in *S*. *cerevisiae*, 16 UBC proteins are found, whereas in human, 40 E2s (including both active E2 proteins and inactive E2 variants) are described [9]. In sequenced algal genomes, 12, 18, and 19 E2 enzymes are encoded in *Ostreococcus tauri*, *Micromonas sp*. *RCC299*, and *Chlamydomonas reinhardtii*, respectively (data not shown). A total of 48 UBC domain-containing proteins have been identified in *Arabidopsis* [10], of which three carry thioester-linked UBLs, 2 are Related Ubiqutin (RUB) conjugating enzymes (RCE1, At4g36800 and RCE2, At2g18600), and one is a SUMO-conjugating enzyme (AtSCE1, At3g57870), so while these UBL-specific enzymes function as E2s, they are not ubiquitin E2s. Eight other UBC proteins lack the Cys active site, referred to as ubiquitin-conjugating enzyme variants (UEVs), are not active by themselves, leaving 37 potential E2s [11]. Kraft et al. [12] classified 37 *Arabidopsis* E2s into 14 groups based on detailed sequence homology analyses. In the rice genome, 48 genes encoding Ub-conjugating (UBC) fold-containing putative E2 proteins were classified into 15 groups [13]. The number of E2s increases with the developmental complexity of the organisms during evolution [14]. Indeed, eukaryotic evolution has also been associated with an increase in the number and diversity of E3s and DUBs [15]. It is now generally believed that genetic expansion and subsequent acquisition of novel molecular functions are the fundamental processes that drive biological diversity [16].

Compared to the extensive studies on E3s, functional studies on E2s in higher plants are limited. Polyubiquitylation through a noncanonical Lys63 chain has been reported, and is required for error-free DNA damage tolerance (or postreplication repair) in plants. Wen et al. [17] cloned and functionally characterized *Arabidopsis UBC13* genes, which functionally complement the yeast *UBC13* null mutant for spontaneous mutagenesis and sensitivity to DNA damaging agents, suggesting the existence of an error-free DNA damage tolerance pathway in plants. Lately, the same research group isolated and characterized four *Arabidopsis UEV1* genes that form a stable complex with AtUBC13, indicating that the UCB13-UEV complex may be involved in DNA repair and damage tolerance [18]. In addition, *AtUBC13* has been implicated in *Arabidopsis* epidermal cell differentiation and iron deficiency responses [17, 19]. COP10 is an E2 enzyme variant lacking a Cys residue that is important for ubiquitin conjugation and necessary for COP10-mediated protein degradation in *Arabidopsis* [20]. COP10 forms protein complex with other E2s, UV-damaged DNA-binding protein 1A (DDB1A), and de-etiolated 1 (DET1), and the resulting complex plays a crucial role in COP1-mediated photo-morphogenesis and is involved in plant tolerance response against UV-B [21]. *AtUBC2* is involved in tolerance response to UV stress, as well as activation of a floral repressor gene [22]. Ectopic expression of E2 gene from wild rice *OgUBC1* confers resistance to UV-B radiation and *Botrytis* infection in *Arabidopsis* [23]. A tomato UBC13-type homologous protein, FNI3, is involved in the regulation of the immune response [24]. Recent reports have shown that tomato-specific E2 regulates fruit ripening [25]. Two classes of E2s (E2-C and UBC4) capable of contributing to APC-dependent protein ubiquitylation *in vivo* during the cell cycle have been reported [26]. *Arabidopsis* encodes two genes belonging to the E2-C gene family (i.e., *UBC19* and *UBC20*), and these proteins functionally replace its yeast ortholog for protein degradation during mitosis, indicating that AtUBC19/20 E2s play a key function in the cell cycle [26]. AtUBC32 is an ERAD component that functions in brassinosteroid-mediated salt stress tolerance [27], whereas AtUBC21 (AtPEX4) is specialized for ubiquitination in peroxisome maintenance [28]. While ubiquitination promotes protein degradation, SUMOylation largely regulates protein interactions [29]. There are at least two distinct and functional SUMO E2 conjugases in *Caenorhabditis reinhardtii*. One (*CrUBC9*) is involved in essential stress-induced SUMOylations, and one (*CrUBC3*) is involved in housekeeping SUMOylation [30]. RUB is an evolutionary conserved 76-amino-acid protein that is closely related to ubiquitin, and its conjugating enzyme 1 (RCE1) has been identified in *Arabidopsis* based on its homology to human UBC12 [31]. RUB conjugation of AtCUL1 affects the function of SCF E3s, which are required for auxin response [32]. PHO2 encodes a putative E2 conjugase, UBC24, which interacts with NLA protein, an E3 ubiquitin ligase, and the NLA-UBC24 complex polyubiquitinates and disrupts the high-affinity Pi transporter, PT2, to regulate Pi homeostasis/signaling in plants [33]. These results suggest that plant E2s play various important roles during plant growth and response to stress.

Maize (*Zea mays L*.) is an important cereal crop and is the staple food for most people around the world. Under natural conditions, high salinity, drought, and cold are the major environmental stresses experienced by maize plants [34, 35]. The completion of the maize genome sequencing project in 2009 has facilitated in the generation of maize models that have been employed in investigating basic biological processes [36]. The complex history of genome duplications and chromosomal rearrangements in maize provides an opportunity to study gene family expansion patterns over the course of genome evolution [37]. To date, information on the *UBC* genes of maize has not been report. The present study provides comprehensive information on the genomic structures, chromosomal locations, sequences homologies, and expression patterns of 75 UBC-containing proteins related to maize development and its response to abiotic conditions (drought, cold, or salt stress). The distinct spatiotemporal expression patterns of *ZmUBC* genes and their differential responses to abiotic stress provides clues for the functional characterization of *UBC* genes involved in maize development and stress response. The expression patterns of each gene, together with the information of orthologs identified from other species, were used in generating inferences on the potential function of *UBC* genes in maize. Our study on UBC protein families in maize has provided valuable genomic resources for future evolutionary, biochemical, and physiological studies on maize.

## Materials and Methods

### 2.1 Identification and bioinformatics analysis of candidate genes

To identify potential members of the maize UBC protein family, published *Arabidopsis*, rice, and yeast UBC protein sequences were retrieved and used as queries in BLASTP searches against the database of the Maize Genetics and Genomics Database (MaizeGDB, http://www.maizegdb.org/) and Phytozome (http://bioinformatics.psb.ugent.be/plaza/versions/plaza/) [38]. The identified putative ZmUBC proteins were designated as ZmUBC1 to ZmUBC75 for convenient discussion of the results. All candidate maize UBC protein sequences were examined using domain analysis programs, Simple Modular Architecture Research Tool (SMART; http://smart.emblheidelberg.de/) [39], Protein family (Pfam; http://pfam.sanger.ac.uk/) [40], and INTERPRO (http://www.ebi.ac.uk/interpro/) [41]. Information on *ZmUBC* genes, including chromosomal location, coding sequence (CDS), exons and introns number, open reading frame (ORF) and amino acid (AA) lengths, was obtained from the maize B73 sequencing database. The molecular weight and theoretical isoelectric point (PI) of the maize UBC proteins were investigated using ExPASy online tools (http://expasy.org/tools/). The exon-intron organization of *ZmUBC* genes was determined by comparing the CDS of its corresponding genomic sequences using the Gene Structure Display Server (GSDS) software (http://gsds.cbi.pku.edu.cn/) [42]. The putative localization of all candidate ZmUBC proteins was analyzed by using TargetP 1.1 (http://www.cbs.dtu.dk/services/TargetP/) [43], WoLF PSORT (http://www.genscript.com/psort/wolf_psort.html), and Plant-mPLoc (http://www.csbio.sjtu.edu.cn/cgi-bin/PlantmPLoc.cgi) [44].

### 2.2 Analysis of phylogenetic relationships and gene duplication

All DNA and amino acid sequence alignments were performed with the MUSCLE program using default parameters [45]. We excluded ambiguously aligned sequences to produce an alignment of 139 amino acid characters for subsequent maximum likelihood (ML) and neighbor joining (NJ) analyses. Phylogenetic trees were constructed with MEGA (Version 6.0; http://www.megasoftware.net/) using the neighbor-joining (NJ) method, and a bootstrap test conducted with 1,000 iterations to test the significance of the nodes [46]. ML analysis was performed on the PhyML web server [47] using the LG substitution model with discrete gamma and four categories. Representations of the calculated trees were constructed using TreeView (Version 1.6.6; http://taxonomy.zoology.gla.ac.uk/rod/treeview). For detection of segmental and tandem duplications, paralogs were regarded as tandem duplicated genes provided two *ZmUBCs* were separated by eight or fewer gene loci according to the maize B73 genome annotation [48]. Paralogs were designated as segmentally duplicated genes when these were located on duplicated chromosomal blocks as previously described by Wei et al.[49].

### 2.3 Plant material and stress treatment

Seeds of maize inbred line B73 were used in the present study. The B73 seeds were surface sterilized, washed with sterile water, and germinated in Petri plates in a greenhouse at 28 ± 2°C, with a photoperiod of 14 h light and 10 h dark. Then, the seedlings were transferred to a nutrient solution (half-strong modified Hoagland’s), the pH of the nutrient solution was adjusted to 5.6, and the nutrient solution was changed every 3 days. All environmental treatments were conducted when uniform-sized seedlings developed three fully opened trifoliate leaves (approximately three weeks after sowing). For cold treatment, seedlings were placed at 4°C for 1 h, 6 h, and 24 h, and maize seedlings were collected at different time points for RNA isolation. For drought treatment, three-week-old maize seedlings were treated with 20% PEG6000 and harvested at time points of 1 h, 6 h, and 24 h after treatment. For salt stress treatment, seedlings were subjected to 200 mM NaCl and harvested at time points of 1 h, 6 h, and 24 h after treatment. In all cases, parallel and untreated plants at the same stage were used as controls. The experiments were performed in triplicate. Student’s *t*-test analysis between untreated seedlings and stress-inoculated plants was performed to identify differential expression patterns of the *ZmUBC* family genes. The leaves of the seedlings were harvested after treatment, flash-frozen in liquid nitrogen, and stored at -80°C until RNA isolation.

### 2.4 RNA isolation and expression analysis

Total RNA was extracted from 0.1 g of tissue using the TRIzol reagent (Invitrogen, Carlsbad, CA, USA), following the manufacturer's instructions. RNA concentrations were determined by using a NanoDrop ND-1000 UV-vis spectrophotometer (NanoDrop Technologies, Inc., Wilmington, USA), and RNA integrity was assessed on a 1% (w/v) agarose gel. Reverse transcription reactions were performed using 5 μg of RNA by M-MLV reverse transcriptase (Takara Bio Inc., Otsu, Japan), following the manufacturer's instructions, after incubation with RNase-free DNase I. Semi-quantitative reverse transcription PCR (RT-PCR) was performed as described elsewhere [50], the PCR conditions were as follows: 94°C for 4 min, followed by 26 cycles of a stepped program (94°C for 40 s, 58°C for 40 s, and 72°C for 30 s), and terminated by an extension at 72°C for 10 min. The maize *actin 1* gene was used as reference. For quantitative real-time PCR, total RNAs were first extracted from various maize samples, following the instructions provided with the TRIzol reagent (Takara). Reverse transcription reactions were performed using 5 μg of RNA with the PrimeScript RT reagent kit with gDNA Eraser (Takara), according to the supplier’s manual. Real-time PCR was performed with a Bio-Rad real-time thermal cycling system (LightCycler 480) using SYBR-green to assess gene expression levels. Each reaction consisted of 10 μL of 2 ×SYBR Premix Ex Taq^™^ II (Takara), 2.0 μL of each cDNA sample, and 400 nM of a gene-specific primer, in a final volume of 20 μL. The primers used are listed in S1 Table. The reaction conditions were as follows: pre-incubation at 94°C for 5 min, followed by 40 cycles at 94°C for 10 s, 58°C–63°C for 20s, 72°C for 30 s. After amplication was complete, a melting curve was obtained by holding at 95°C for 5 s and then at 65°C for 15 s, followed by heating slowly at 0.1°C/s to 95°C. Quantification of results was obtained by using a CFX96^™^ Real-Time PCR Detection System (Bio-Rad Laboratories, Inc., USA). The specificity of the reactions was verified by melting curve analysis. The relative mRNA level for each gene was calculated as △△*C*_T_ values relative to that of untreated seedlings. Maize *actin 1* gene was used as internal control for normalization. cDNAs from three biological samples were used for analysis, and all the reactions were run in triplicate. In the comparative expression analysis of *ZmUBCs*, genes that were up- or downregulated by at least two-fold were considered differentially expressed.

## Results and Discussion

### Identification of maize genes encoding UBCc-E2 proteins

In the present study, we used the previously reported E2 proteins from *Arabidopsis*, rice, and yeast as BLAST queries to search for maize E2s in the MaizeGDB and Phytozome10.3 databases. A total of 82 genes encoding UEVs were detected in maize. E2s for conjugating Rub (family UBC12) and SUMO (family UBC9) were also identified. After the redundant sequences were removed, a total of 80 sequences were obtained. Further scanning of the 80 sequences for the UBCc domain by motif scan using SMART, Pfam, or INTERPRO searches with filter off by default setting reduced the total number of maize UBC protein family members to 75. For convenience, the 75 *ZmUBC* genes were named *ZmUBC-01* to *ZmUBC-75* based on their order on the chromosomes, from chromosomes 1 to 10. The information of these 75 UBC-encoding genes, including TIGR locus, chromosome location, ORF, intron, size, PI, MW (kDa), and putative localization of each protein is shown in Table 1. The size of the deduced UBC proteins varied markedly, ranging from 133 amino acids (*ZmUBC-29*) to 1,102 amino acids (*ZmUBC-56*), its corresponding molecular mass ranged from 12.49 kDa to 121.98 kDa, and its predicted isoelectric point was between 4.09 (ZmUBC-02) and 11.07 (ZmUBC-07). In addition, the subcellular localization analysis showed that most of the ZmUBC proteins were located in the nucleus, only a few ZmUBC proteins had functions related to the Cytoplasm, chloroplast, mitochondrion, or endoplasmic reticulum. These findings suggested that different UBC proteins functioned in different micro-environments. These result were partially confirmed by previous reports involving the AtPEX4 protein, which was localized in the peroxisome, AtUBC32 in the ER, and AtPHO2 in the endomembrane [27, 28, 51], GmUBC2 in both the cytosol and nucleus, and AtCOP10, a UEV that has been localized to the nucleus [52].

**Table 1:** The information of ZmE2 gene family.

Gene name	Gene locus	Chromosome Location	ORF(bp)	Size(aa)	PI	MW(KDa)	Intron	Putative localizatin
***ZmUBC-01***	GRMZM2G070047	Chr1: 4,981,301–4,984,643	483	160	8.43	18.03	4	Nucleus
***ZmUBC-02***	GRMZM2G150867	Chr1: 10,590,399–10,593,290	552	183	4.09	19.16	4	Nucleus
***ZmUBC-03***	GRMZM5G824629	Chr1: 49,614,813–49,617,825	945	314	5.52	34.45	4	Nucleus
***ZmUBC-04***	GRMZM2G312693	Chr1: 83,176,776–83,179,023	483	160	8.43	17.99	4	Nucleus
***ZmUBC-05***	GRMZM2G007381	Chr1: 180,012,788–180,017,472	486	161	7.72	18.25	5	Nucleus
***ZmUBC-06***	GRMZM2G053764	Chr1: 236,088,923–236,128,384	1113	370	4.60	41.69	10	Nucleus
***ZmUBC-07***	GRMZM2G116840	Chr1: 256,367,147–256,407,916	417	138	11.07	15.44	1	Nucleus
***ZmUBC-08***	GRMZM2G022859	Chr1:267,922,012–267,928,571	486	161	5.13	18.36	4	Nucleus
***ZmUBC-09***	GRMZM2G120674	Chr1: 286,845,415–286,850,582	459	152	5.37	17.30	4	Nucleus
***ZmUBC-10***	GRMZM2G102471	Chr2: 2,803,488–2,806,329	447	148	7.72	16.51	3	Nucleus
***ZmUBC-11***	GRMZM2G038851	Chr2: 15,245,261–15,247,000	480	159	9.12	17.73	4	Nucleus
***ZmUBC-12***	GRMZM2G341089	Chr2: 15,269,254–15,273,461	480	159	5.25	12.57	2	Nucleus
***ZmUBC-13***	GRMZM2G016176	Chr2: 161,497,123–161,501,579	762	253	10.16	28.54	10	Nucleus
***ZmUBC-14***	GRMZM2G000601	Chr2: 172,703,543–172,707,425	447	148	6.40	16.66	1	Nucleus
***ZmUBC-15***	GRMZM2G113396	Chr2: 206,899,426–206,903,057	573	190	9.58	20.84	3	Nucleus
***ZmUBC-16***	GRMZM2G022206	Chr2: 233,793,739–233,798,436	459	152	5.64	17.34	3	Nucleus
***ZmUBC-17***	GRMZM2G010460	Chr3: 1,684,241–1,688,895	1122	373	8.39	41.93	6	Nucleus
***ZmUBC-18***	GRMZM2G123519	Chr3: 1,726,294–1,729,668	1155	384	4.91	43.51	4	Nucleus
***ZmUBC-19***	GRMZM2G086583	Chr3: 24,458,611–24,460,536	804	267	6.71	30.29	3	Nucleus
***ZmUBC-20***	GRMZM2G002830	Chr3: 41,472,011–41,473,369	552	183	5.17	19.57	2	Nucleus
***ZmUBC-21***	GRMZM2G434519	Chr3: 55,353,651–55,355,715	525	174	6.96	19.60	3	Nucleus
***ZmUBC-22***	GRMZM2G018447	Chr3: 93,187,188–93,205,169	486	161	7.72	18.23	5	Nucleus
***ZmUBC-23***	GRMZM5G866947	Chr8: 170,631,089–170,636,009	447	148	7.71	16.49	3	Nucleus
***ZmUBC-24***	GRMZM2G007300	Chr3: 181,446,796–181,451,566	510	169	5.05	18.98	5	Nucleus
***ZmUBC-25***	GRMZM5G862131	Chr3: 209,846,496–209,850,012	462	153	6.74	17.20	7	Nucleus
***ZmUBC-26***	GRMZM5G814314	Chr3: 212,436,396–212,440,285	444	147	7.72	16.55	3	Nucleus
***ZmUBC-27***	GRMZM2G157605	Chr3: 223,745,568–223,748,681	1734	577	8.57	63.23	5	Nucleus
***ZmUBC-28***	GRMZM2G106143	Chr4: 50,730,408–50,737,299	954	317	9.74	35.90	11	Cytoplasm
***ZmUBC-29***	GRMZM2G461533	Chr4: 145,324,353–145,325,491	402	133	8.42	14.90	4	Nucleus
***ZmUBC-30***	GRMZM2G161545	Chr4: 145,368,230–145,371,847	474	157	8.69	17.80	3	Nucleus
***ZmUBC-31***	GRMZM2G027378	Chr4: 220,508,652–220,512,101	447	148	6.42	16.77	3	Nucleus
***ZmUBC-32***	AC233922.1_FGT008	Chr4: 238,041,057–238,042,180	447	148	7.70	16.51	2	Nucleus
***ZmUBC-33***	GRMZM2G007260	Chr5: 4,495,065–4,499,934	618	205	6.08	13.54	4	Nucleus
***ZmUBC-34***	GRMZM2G090172	Chr5: 10,931,101–10,936,807	501	166	5.40	18.93	4	Cytoplasm. Nucleus
***ZmUBC-35***	GRMZM2G148130	Chr5: 13,646,747–13,649,731	495	164	6.02	14.73	3	Nucleus
***ZmUBC-36***	GRMZM2G146374	Chr5: 31,648,146–31,651,787	624	207	4.49	23.32	3	Nucleus
***ZmUBC-37***	GRMZM2G115939	Chr5: 56,740,173–56,751,799	555	184	8.67	20.66	4	Nucleus
***ZmUBC-38***	GRMZM2G466265	Chr5: 70,756,337–70,760,939	1149	382	9.11	43.33	6	Nucleus
***ZmUBC-39***	GRMZM5G828302	Chr5: 188,118,457–188,127,477	474	157	8.69	17.80	3	Nucleus
***ZmUBC-40***	GRMZM2G411771	Chr5: 193,685,680–193,722,957	984	327	4.86	36.65	5	Cytoplasm
***ZmUBC-41***	GRMZM2G147579	Chr5:217,470,848–217,472,628	1437	478	9.65	52.13	0	Nucleus
***ZmUBC-42***	GRMZM2G110983	Chr6: 66,201,455–66,205,271	537	178	4.82	12.82	4	Nucleus
***ZmUBC-43***	GRMZM2G102421	Chr6: 87,278,503–87,284,723	552	183	8.36	20.63	4	Nucleus
***ZmUBC-44***	GRMZM5G895435	Chr6: 94,713,649–94,717,995	762	253	8.98	27.47	4	Nucleus
***ZmUBC-45***	GRMZM2G116919	Chr6:107,362,893–107,368,461	720	239	9.01	26.91	8	Nucleus
***ZmUBC-46***	GRMZM2G156517	Chr6: 110,386,088–110,389,342	447	148	7.68	16.52	3	Nucleus
***ZmUBC-47***	GRMZM2G012052	Chr6: 151,770,259–151,778,529	510	169	5.04	18.97	4	Nucleus
***ZmUBC-48***	GRMZM2G085849	Chr6: 163,835,366–163,840,407	1524	507	5.61	56.87	7	Nucleus
***ZmUBC-49***	GRMZM2G381709	Chr6: 163,843,112–163,847,600	2616	871	4.77	96.37	7	Nucleus
***ZmUBC-50***	GRMZM2G007122	Chr6: 164,260,122–164,263,444	465	154	6.67	17.34	7	Nucleus
***ZmUBC-51***	GRMZM2G433968	Chr7: 12,311,212–12,312,732	534	177	4.73	19.29	2	Nucleus
***ZmUBC-52***	GRMZM2G173756	Chr7: 79,006,728–79,011,032	447	148	6.40	16.68	1	Nucleus
***ZmUBC-53***	GRMZM2G056501	Chr7: 82,007,091–82,010,522	486	161	6.96	18.30	3	Nucleus
***ZmUBC-54***	GRMZM2G072506	Chr7: 160,242,028–160,246,175	555	184	8.53	19.84	3	Nucleus
***ZmUBC-55***	GRMZM2G007057	Chr8: 3,195,388–3,196,814	765	254	5.36	19.5	2	Nucleus
***ZmUBC-56***	GRMZM2G078360	Chr8: 10,926,318–10,939,316	3309	1102	4.55	121.98	6	Nucleus
***ZmUBC-57***	GRMZM2G122003	Chr8: 28,846,608–28,852,930	1317	438	5.14	49.63	5	Nucleus
***ZmUBC-58***	GRMZM2G027546	Chr8: 67,380,589–67,387,931	1563	520	5.42	58.37	7	Nucleus
***ZmUBC-59***	GRMZM2G015287	Chr8: 68,928,768–68,932,536	462	153	6.74	17.21	7	Nucleus
***ZmUBC-60***	GRMZM2G007276	Chr8: 116,776,837–116,781,561	510	169	6.28	12.49	3	Nucleus
***ZmUBC-61***	GRMZM2G132759	Chr8: 119,039,106–119,043,451	510	169	7.71	16.50	3	Nucleus
***ZmUBC-62***	GRMZM2G086088	Chr8: 148,200,019–148,203,135	444	147	7.72	16.55	3	Nucleus
***ZmUBC-63***	GRMZM2G115828	Chr8: 150,010,557–150,014,289	468	155	6.74	17.19	7	Nucleus
***ZmUBC-64***	GRMZM2G134176	Chr8: 160,396,937–160,401,812	594	197	4.77	21.39	4	Nucleus
***ZmUBC-65***	GRMZM2G085600	Chr8: 169,009,649–169,013,585	510	169	4.40	16.43	5	Nucleus
***ZmUBC-66***	GRMZM2G177276	Chr9: 11,703,081–11,707,342	720	239	9.01	26.99	8	Nucleus
***ZmUBC-67***	GRMZM2G464572	Chr9: 56,902,708–56,907,334	2616	871	4.74	96.37	7	Nucleus
***ZmUBC-68***	GRMZM2G153924	Chr9: 105,689,843–105,694,388	759	252	9.22	27.38	4	Nucleus
***ZmUBC-69***	GRMZM2G163398	Chr9: 125,296,709–125,299,946	483	160	7.76	18.07	4	Nucleus
***ZmUBC-70***	GRMZM2G121303	Chr9: 135,149,922–135,154,003	552	173	5.40	18.72	2	Nucleus
***ZmUBC-71***	GRMZM2G440918	Chr9: 143,394,526–143,397,331	957	318	9.03	35.37	3	Nucleus
***ZmUBC-72***	AC149818.2_FGT006	Chr9: 152,442,986–152,445,781	552	183	4.27	20.87	5	Nucleus
***ZmUBC-73***	GRMZM2G063931	Chr9: 153,779,802–153,784,716	483	160	8.42	18.01	4	Nucleus
***ZmUBC-74***	GRMZM2G109582	Chr10: 21,820,190–21,821,376	1053	350	6.16	38.01	0	Nucleus
***ZmUBC-75***	GRMZM2G146142	Chr10: 62,900,930–62,907,699	1146	381	9.99	42.86	8	Nucleus

### Chromosomal distribution and gene duplication of maize UBCc-E2 genes

**Fig 1:**
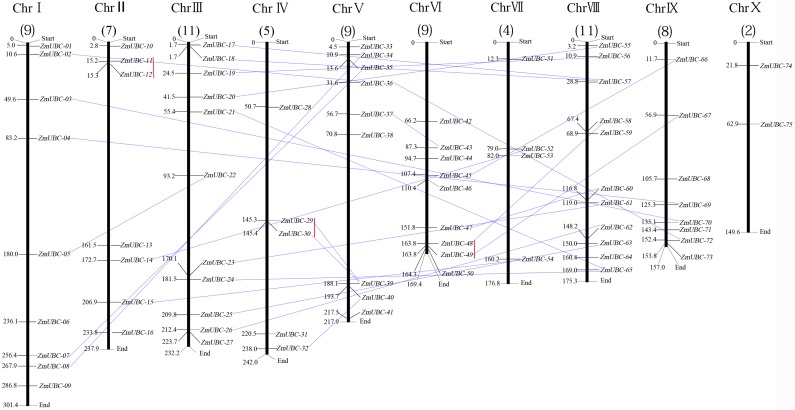
Chromosomal and gene duplication events involving *ZmUBC* genes. The chromosomal position of each UBC-encoding gene was mapped to the maize genome. The chromosomal number is indicated at the top of each chromosome. The number below represented the number of UBC-encoding genes in each chromosome. The segmental duplication genes are connected by a blue straight line. The tandemly duplicated genes are linked by a red straight line. Chromosomal distances are in Mbp.

Chromosomal location analyses showed that 75 *ZmUBC* genes were distributed across 10 chromosomes (Fig 1). The distribution of the *ZmUBC* genes was highly variable; only 2 genes were detected on chromosome X, 4 genes was found on chromosome VII, 5 genes was found on chromosome IV, 7 genes were localized to chromosome II, 8 genes on chromosome IX, whereas chromosomes I, V andVI harbored 9 *ZmUBC* genes. The largest number of *ZmUBC* genes was identified on chromosomes III and VIII (11 genes). According to the definition of gene clusters [53], out of 75 maize homeobox genes, 21 gene pairs were assigned to 10 maize linkage groups, whereas the rest were located on scaffolds. Some *ZmUBC* genes were sparsely situated on maize chromosomes, whereas some were densely distribution in other chromosomes.

Gene duplication plays an extremely important role in gene family expansion and protein functional diversification [54]. To explore the contribution of duplication events to this family, we analyzed the occurrence of tandem duplications and large-scale segmental duplications during the evolution of this gene family. Phylogenetic analysis and chromosomal localization of *ZmUBC* genes determined that a total of 24 duplicated maize gene pairs (48 of 75 *ZmUBC* genes, 64%) underwent segmental duplication, and three gene pairs (6 of 75 *ZmUBC* genes, 8%) were involved in tandem duplication (*ZmUBC-11* and *ZmUBC-12*, *ZmUBC29* and *ZmUBC30*, *ZmUBC48* and *ZmUBC49*) (Fig 1). A recent report showed that 15 of 39 (38.5%) *OsUBCs* resulted from segmental duplication events, and four genes (10.2%) from tandem duplication, indicating the different roles of gene duplication in the evolution of E2s between the maize and rice genomes [55]. The three tandemly duplicated *ZmUBC* genes were localized to chromosomes 2, 4, and 6 and grouped into subfamilies UBC9, UBC10, and UBC15, respectively. These findings suggested that gene duplication events, particularly segmental duplications, contributed to the expansion of the *ZmUBC* family in maize.

### Phylogenetic analysis of the *ZmUBC* gene family

**Fig 2:**
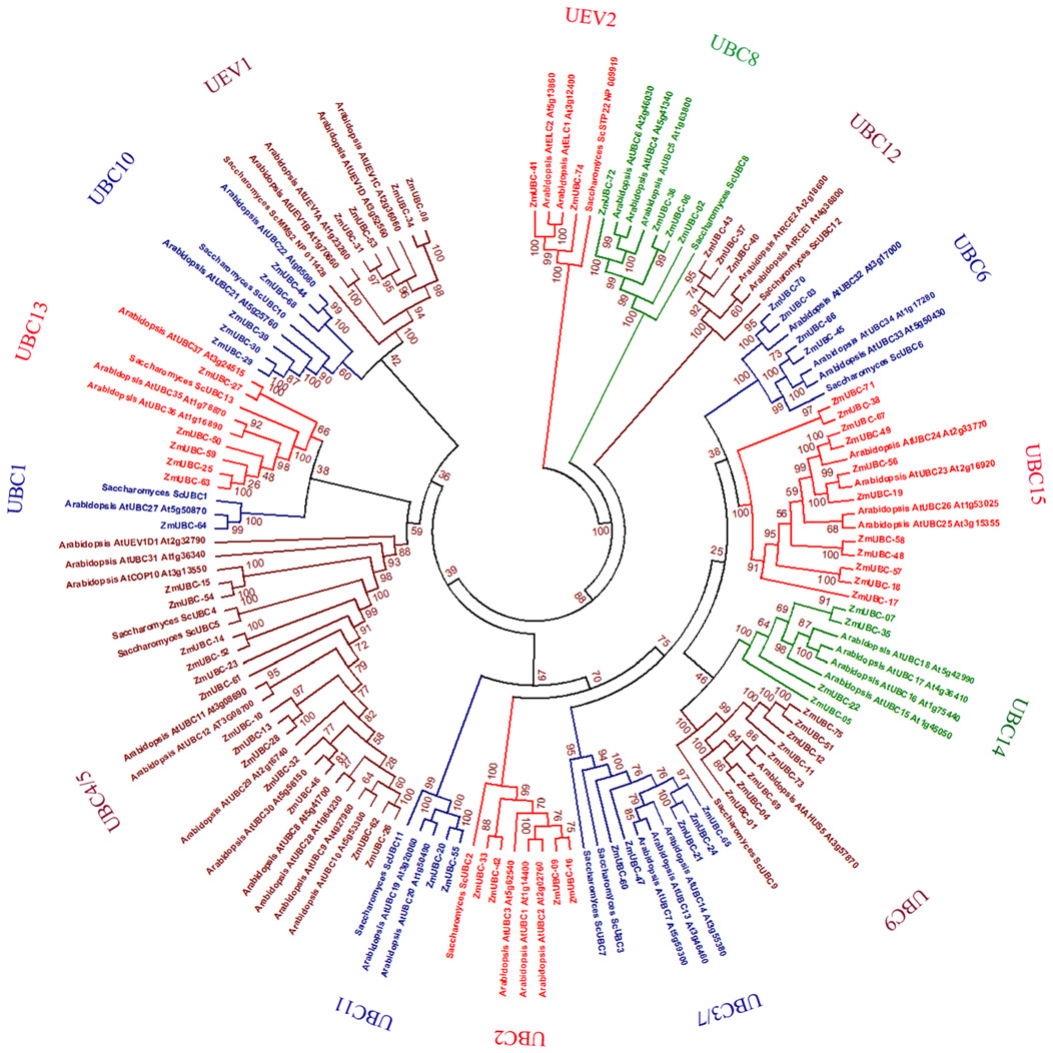
Phylogenetic relationship of various ZmE2s. The ML tree includes 75 UBC proteins from *Zea mays*, 15 from *Saccharomyces cerevisiae*, and 48 from *Arabidopsis thaliana*. The tree shows 15 phylogenetic subgroups depicted in various colors to distinguish diversification of subfamilies into clusters.

To evaluate the evolutionary relationship of the ZmUBC proteins, full-length amino acid sequences of 75 *ZmUBC* genes, 15 UBC-encoding genes from *Saccharomyces cerevisiae*, and 48 UBC-encoding genes from *A*. *thaliana* were subjected to a multiple sequence alignment using MUSCLE and later manually optimized. The alignment was then used for the construction of an unrooted phylogenetic tree using the ML and NJ methods. Fig 2 and S1 Fig shows that based on the consistent topology between ML and NJ trees, except for some tiny differences, the UBC proteins could be further classified into 13 E2 groups and two independent UEV groups as monophyletic clades with at least 50% bootstrap support, with groups UBC9 and UBC12 functioning in SUMO and RUB1 conjugation pathways and COP10. The third UEV group was nested in the UBC4/5 subfamily. The subfamilies were named according to their identity to *S*. *cerevisiae* UBC proteins: UBC1, UBC2, UBC3, UBC4/5, UBC6, UBC3/7, UBC8, UBC9, UBC10, UBC11, UBC12, UBC13, UBC14, UBC15, UEV1 and UEV2. The subfamilies UBC4/5 and UBC3/7 shared two highly identical paralogous yeast genes respectively, according to previous reports we named this two subclades as subfamily UBC4/5 and UBC3/7 [14, 56]. The result is consistent with the findings of previous studies [12]. The analysis shows that all maize UBC proteins have orthologous *Arabidopsis* proteins and green algae proteins (data not shown). These findings also indicated that the highly complex extant genomes and the expansion of *Arabidopsis* UBC-encoding genes were derived from a common ancestor.

Comparison of the number of UBC-encoding genes and its corresponding mRNAs within and between species indicated that both gene duplication and alternative splicing contributed to the genomic complexity and proteomic diversity of the *ZmUBC* genes. The phylogenetic tree shows that UBC4/5 and UBC15 underwent considerable expansion of 13 and 11 E2s, respectively, which comprised the largest subfamily, whereas the clades of UEV2, UBC1, and UBC11 only have one or two UBC proteins in maize. The remaining subfamilies have an intermediate number E2s, ranging from 3 in UBC12 to 8 in UBC9. These findings indicated that each group has a different evolutionary history that finally formed the E2 family in the extant species. Alternative splicing can generate more transcripts from a single gene than the number of genes in an entire genome. The observed high percentages [(human: 19/50 (38%), *Arabidopsis*: 21/48 (43.8%), maize: 52/75 (69.3%)] of alternatively spliced transcripts indicated that maize E2s underwent extensive expansion at the transcriptional level.

### Gene structure and conserved motif distribution analysis

**Fig 3:**
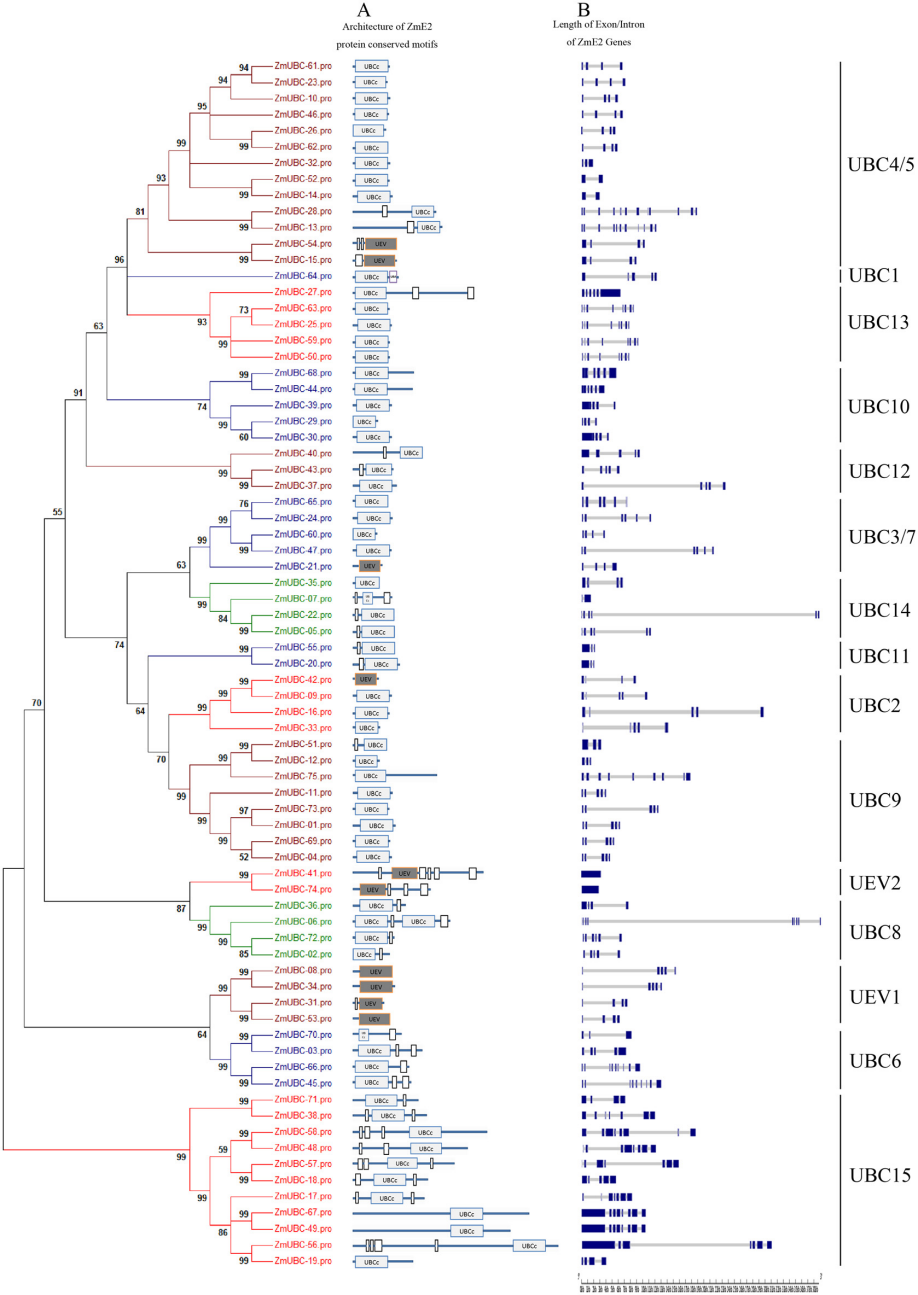
The architecture of conserved protein motifs and intron pattern of ZmE2s. (A) Architecture of conserved protein motifs of UBC proteins. (B) The gene structure is presented by blue exon(s) and spaces between the blue box corresponding to intron (s). The sizes of the exons and intron can be estimated using the horizontal lines.

We then used the SMART program to analyze the conserved motifs in UBC proteins. Fig 3A shows that according to the UBC/UEV domain and the N- or C-terminal structure, the ZmUBC proteins could be further divided into four classes [2, 12, 57]. Class I proteins mainly comprised the UBC/UEV domain, which contained 42 ZmUBC proteins, and those genes contained in all groups except UBC 1, 6, 8, and 11 and UEV 2. Class II proteins harbored the UBC/UEV domain plus a C-terminal extension, which contained 15 UBC proteins, and those genes contained in UBC 4/5, 9, 11, 12, 14, 15, and UEV 1. Class III proteins have the UBC/UEV domain plus an N-terminal extension, which contained 12 ZmUBC proteins, and those genes contained in UBC 1, 6, 8, 13, 15, and UEV2. Class IV proteins have the UBC/UEV domain plus both the N-terminal and C-terminal extension, which consisted of 6 ZmUBC proteins, and those genes contained in UBC 14, UBC 15, and UEV 2.

To better understand the structural diversity of *ZmUBC* genes, its intron/exon arrangements and conserved motifs were also compared. We obtained each gene structure by comparing their ORFs with their corresponding genomic sequences. Table 1 and Fig 3B shows that all the *ZmUBC* genes possessed at least 1 intron. The most common pattern contained 3–4 introns (63% of *ZmUBC*s). In addition, most members within the same subfamily shared the same exon/intron structure. For example, in subfamily UBC 2, *ZmUBC-09*, *ZmUBC-16*, *ZmUBC-33*, and *ZmUBC-42* consisted of four introns, *ZmUBC-20* and *ZmUBC-55* in subfamily UBC11 harbored two introns, and UBC 11, 12, UEV 1, and UEV 2 presented the same exon/intron structure. The members of UBC9, 10, and 13 within the same subfamily shared two kinds of exon/intron structures. For example, in UBC 10, *ZmUBC-29*, *ZmUBC-30*, and *ZmUBC-39* consisted of three introns, and two genes (*ZmUBC-44* and *ZmUBC-68*) harbored four introns. In contrast to the highly conserved structural patterns in the above groups, the members of UBC 4/5, 3/7, 6, 8, 14, and 15 showed a complex distribution of exons and introns, including different pattern subsets within the same phylogenetic group. For instance, in group 4/5, 10 genes harbored less than 3 introns, whereas two genes consisted of more than 10 introns. Overall, most closely related members in the same subfamilies shared a similar exon/intron structure in terms of intron number and exon length.

### The expression pattern of *ZmUBC* genes

**Fig 4:**
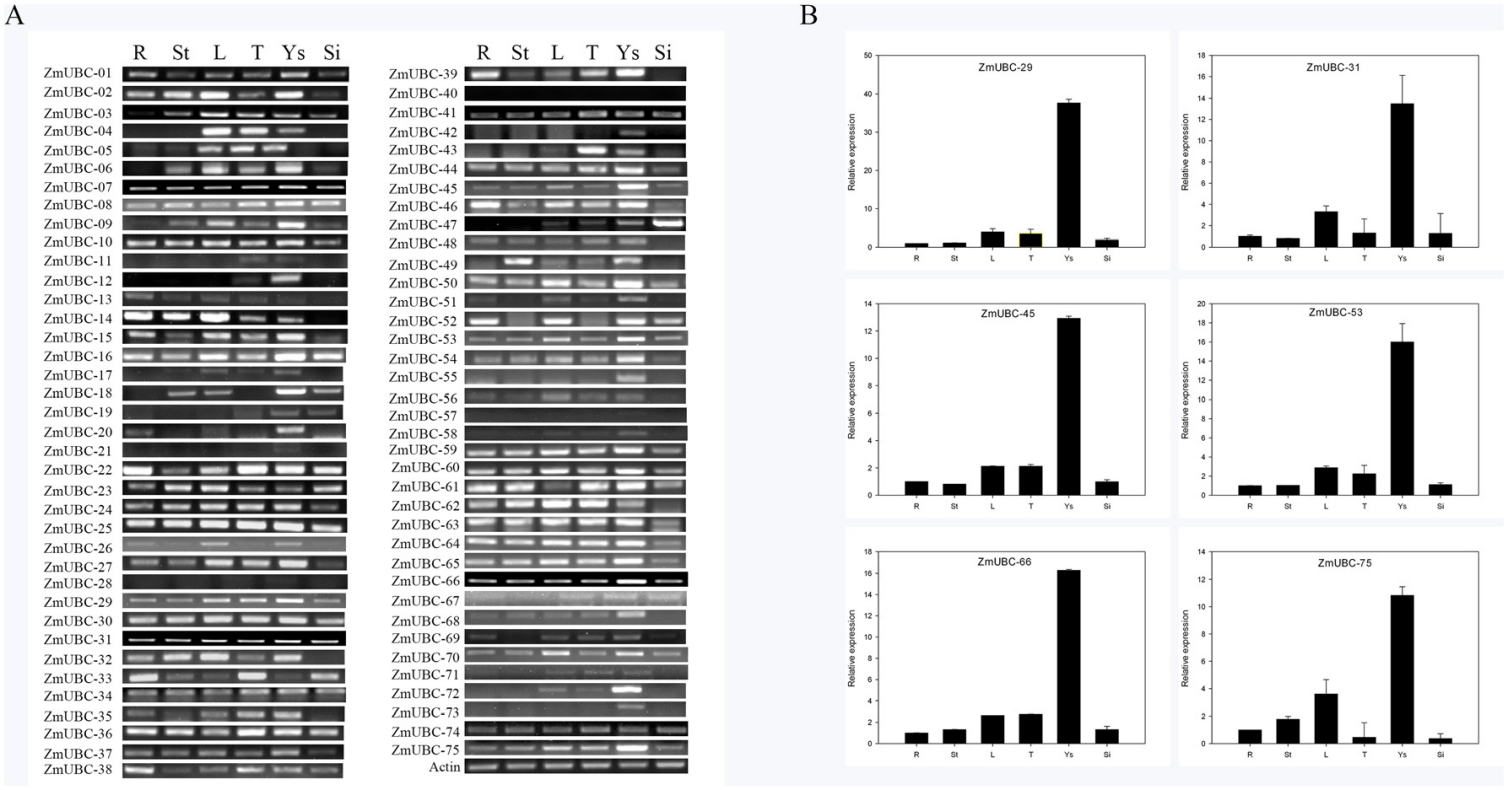
Tissue-specific expression of *ZmUBC* genes. (A) Semi-quantitative RT-PCR analysis of *ZmUBC* genes. (B) RT-qPCR analysis of representative *ZmUBC* genes. The letter R above the column of the expression data refers to root, St represents stem, L indicates leaf, T refers to tassel, Ys indicates young seed, and Si represents silk.

Because gene expression patterns have been examined in relation to gene function [58], the expression of the *ZmUBC* gene in different tissues was investigated. Using a semi-quantitative RT-PCR approach, the mRNA accumulation of each gene in the root, stem, leaf, tassel, young seed (YS), and silk tissues was assessed. Fig 4 shows that among the 75 UBC-encoding genes, 10 genes were constitutively expressed in different tissues at similar levels, and 61 genes were expressed in at least one tissue, whereas the transcript levels of 4 genes were undetectable, suggesting these might be pseudogenes or might be expressed at specific developmental stages, or under special conditions.

By combining these results, we determined that among the 61 *ZmUBC* genes, 4 genes were only detected in one tissue, 3 genes were detected in two tissues, 8 genes in three tissues, 5 genes in four tissues, 11 genes in five tissues, and 30 genes in all tested tissues with tissue-specific expression profiles. Intriguingly, the expression of *ZmUBC-12*, *ZmUBC-20*, and *ZmUBC-72* was only detected in YSs, suggesting that those genes might be involved in the formation and development of fruit. To validate the results of semi-quantitative PCR analysis, RT-qPCR analysis was performed on representative genes. The results indicated that the expression patterns of these genes were in general agreement with the findings of RT-PCR analysis. Six tested genes (*ZmUBC-29/31/45/53/60/75*) showed high expression levels in YS, with an approximately 10-40-fold higher level of expression than that observed in the root. Similar results were also reported in rice. Five rice *UBCs* (*OsUBC13/17/18/26/35*) exhibited either predominant or tissue-specific expression in YR, YL, or ML, and four genes (*OsUBC13/32/33/34*) were highly expressed in leaves [55]. In *Arabidopsis*, *AtUBC1* and *AtUBC2* were ubiquitously expressed in the roots, leaves, flowers, and seedlings, and the double mutant *atubc1-1atubc2-1* showed a dramatically reduced number of rosette leaves and an early-flowering phenotype [22]. However, *OsUBC7* and *OsUBC8*, orthologs of *AtUBC1* and *AtUBC2*, were upregulated in most organs, particularly in YR and ML. Collectively, our results indicated that *ZmUBC* genes played multiple roles in the development of maize.

### Expression profiles of *ZmUBC* genes in response to various stressors

Plants and crops are frequently challenged by abiotic stressors such as salt, drought, and low temperature. Recent studies have suggested that UBC proteins are widely involved in signaling and response to abiotic stimuli [23, 27, 55], although information on the involvement of UBC proteins in stress responses in maize is limited. To investigate the potential roles of *ZmUBC* genes in response to environmental stresses, we examined their expression patterns under salt (200 mM NaCl), drought (20% PEG 6000), and cold (4°C) treatment as described in the Materials and Methods section. Untreated seedlings grown in nutrient solution were used as control seedlings.

**Fig 5:**
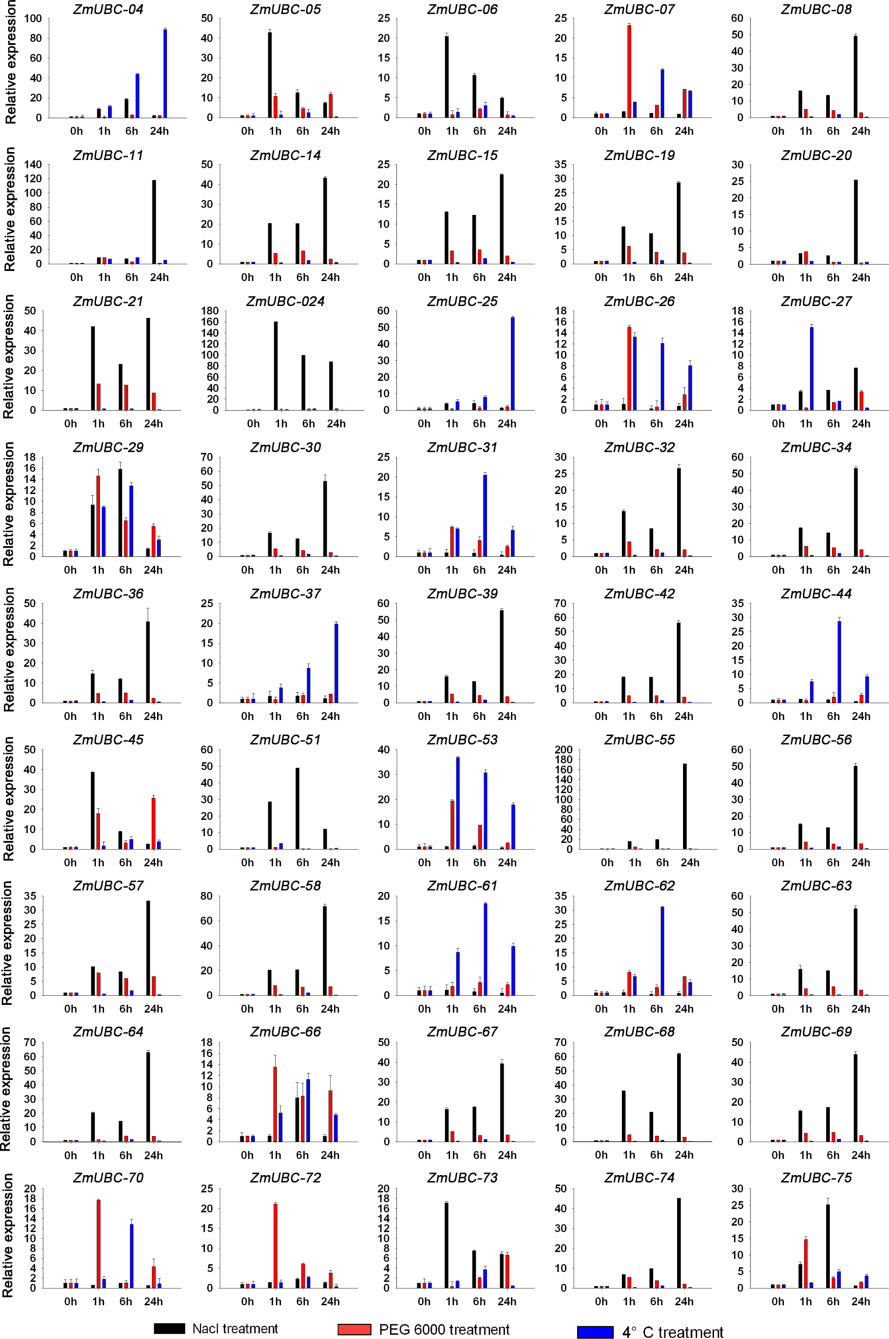
Differential expression levels of *ZmUBC* genes following NaCl, PEG 6000, and 4°C treatment. The X-axis indicates time course/treatment and the Y-axes are scales of relative expression levels. The maize *actin* gene was used as internal control. The presented data are representative of three independent experiments.

Fig 5 and S2 Fig shows that under NaCl treatment, all of the *ZmUBCs* tested, except 14 genes (*ZmUBC-01/07/16/26/31/37/44/46/47/53/61/62/70*), were upregulated by salt stress. Twenty-six genes (*ZmUBC-02/03/04/05/06/09/10/12/18/22/24/25/28/29/33/35/45/48/49/50/51/52/54/65/73/75*) were upregulated at early time points during treatment, downregulated at later time points. Thirty-five genes (*ZmUBC-08/11/13/14/15/17/19/20/21/23/24/27/30/32/34/36/38/39/40/41/42/43/55/56/57/58/59/60/63/64/66/67/68/69/71*) were upregulated throughout the NaCl treatment. The *ZmUBC-08/11/24/30/34/39/42/58/63/64* genes were upregulated by >50-fold at the 24 h time point of the NaCl treatment. By comparing the expression data of each pair of duplicated *ZmUBC* genes, 14 pairs of duplicated genes (*ZmUBC-5/22; ZmUBC-08/34*; *ZmUBC-19/56; ZmUBC-20/55; ZmUBC29/30/39*; *ZmUBC21/24/65*; *ZmUBC48/58*; *ZmUBC25/63*; *ZmUBC26/62*; *ZmUBC15/54*; *ZmUBC14/52*; *ZmUBC-04/69*; *ZmUBC49/67*; and *ZmUBC45/66*) in salt stress showed a similar expression pattern. However, 8 pairs of duplicated genes (*ZmUBC-02/36/72*, *ZmUBC-03/70*; *ZmUBC07/35*; *ZmUBC-17/18/57; ZmUBC-23/61; ZmUBC-32/40; ZmUBC37/43*; and *ZmUBC47/60*) showed completely differently expression profiles during salt stress, which can be attributed to lack of intense selection pressure and need for diversification.

For PEG6000 treatment, 34 genes (*ZmUBC-06/08/11/14/15/18/22/26/28/29/30/31/32/34/36/39/40/41/42/47/53/55/56/57/58/62/63/65/67/68/69/70/72/74/75*) were upregulated during early time points, and then downregulated at later time points. The expression levels of 22 genes (*ZmUBC-02/03/05/07/09/10/13/16/17/19/20/21/23/27/35/44/45/46/48/64/66/73*) were continuously increased throughout the time course. On the other hand, 19 genes (*ZmUBC-01/04/12/17/* 24/25/33/37/38/43/49/50/51/53/54/59/60/61/71) were not responsive to PEG-monitored drought stress. For the 4°C treatment, 28 genes (*ZmUBC-03/07/09/10/11/16/18/26/27/28/29/31/40/41/44/45/50/51/52/53/57/61/62/66/70/75*) were upregulated during the early time points of the treatment, then downregulated at the later time points. The expression level of 42 genes(*ZmUBC-01/02/05/06/08/12/13/14/15/17/19/20/21/22/23/24/30/32/34/35/36/38/39/42/43/47/54/55/56/58/59/60/63/65/67/68/69/71/72/73/74*) did not change with the 4°C treatment, whereas five genes (*ZmUBC-04/25/33/37/46*) were upregulated with the 4°C treatment. *ZmUBC-25* was upregulated by >50-fold at the 24 h time point during cold treatment.

Similar to salt stress, 18 and the 14 pairs of duplicated genes under drought and cold stress, respectively, showed a similar expressional pattern, whereas 8 and 14 pairs of duplicated genes under drought and cold stress, respectively, showed drastically differential expression profiles. In addition, a total of 6 *ZmUBCs* (*ZmUBC-07*, *ZmUBC-26*, *ZmUBC-45*, *ZmUBC-53*, *ZmUBC-70*, and *ZmUBC-75*) were markedly upregulated at least by two stimuli, and two genes, *ZmUBC29* and *ZmUBC65*, was strongly induced by three stimuli, indicating that these genes played a complex role in response to abiotic stress. We concluded that ZmUBCs played an essential role in response to abiotic stresses. This conclusion was supported by the previously reported plant homologous genes such as *AtUBC32*, *AtUBC24*, *OgUBC1*, *CmUBC2*, and *VrUBC1* [23, 27, 51, 55], which were identified as salt-responsive genes and regulate salt tolerance in plants.

## Discussion

Ubiquitin-conjugating enzymes are an integral component of the ubiquitin proteasome system that is involved in very important roles during plant development and growth. Despite the potential functional significance of UBC members, only a few UBC family members have been described in higher plants. Maize (*Z*. *mays L*.) is one of the most important cereal crops and a plant model for investigations in genetics, evolution, and domestication. Drought, cold, and salt stresses can all be harmful to plants by causing cellular desiccation, and plants combat these three stresses using a common mechanism [55]. This background knowledge prompted us to identify the full complement and expression profile of this important gene family during development and under abiotic stresses in maize.

In the present study, a comprehensive set of 75 nonredundant UBC-encoding genes (including 69 E2s and 6 UEVs) were identified and characterized from the current version of the maize (B73) genome. Exploration for the full complement of UBC proteins in *Arabidopsis* and rice genomes resulted in the identification of 45 and 48 genes, respectively [12, 55]. A total of 52 nonredundant E2 genes have also recently been identified in tomato [25]. The observed higher number of UBC-encoding genes in maize can not only be explained by its larger genome size (~2,300 Mb) compared to that of *Arabidopsis* and rice (~125 Mb and ~389 Mb), as a similar number of UBC-encoding genes exist in these species (48 and 45 genes, respectively), although the size of the rice genome is ~3.7 times larger than that of *Arabidopsis*. Gene duplication events play a significant role in the amplification of gene family members in the genome [55]. Research has estimated that the fraction of retained paralogs is 72% in maize, which occurred over the course of the past 11 million years [59]. The expansion mechanism of the *ZmUBC* gene family was analyzed to understand gene duplication events. In our study, a total of 24 sister gene pairs of maize UBC proteins were determined to be involved in segmental duplications by shared phylogenetic clade combinations within the same groups and by locations within the segmental duplicated blocks. Three maize UBC genes involved in tandem duplication were localized to chromosomes 2, 3, and 4, respectively, forming a single cluster. These results suggest that segmental duplications are the main contributor to the expansion of the maize UBC family. Therefore, it can be inferred that the expansion of the *ZmUBC* gene family might not solely rely on the independent duplication of individual sequences and thus might also be the consequence of segmental chromosomal duplication and rearrangement events. An increasing number of studies has shown that segmental duplications are largely responsible for the expansion of maize gene families, which include the CCCH, HD-Zip HSF, bZIP, and PRX gene families [60–63].

The phylogenetic analysis categorized all the ZmUBCs into 13 discrete E2 groups and 2 UEV groups. Our classification resulted in similar clusters, similar to that described in previous studies involving *Arabidopsis* [12], tomato [25], and rice [55], although with minimal differences. For example, two genes, *AtUBC21* and *AtUBC22*, were grouped as a subfamily with weak bootstrap support (60%) in our study, whereas their orthologs were divided into two subfamilies in previous results [12, 25, 55]. The congruity of tree topology in different studies indicated the reliability of our clade- and subgroup designations. The phylogenetic tree showed obvious differences in numbers among subfamilies. Subfamilies UBC4/5, UBC15, and UBC9 have 13, 11, and 8 members, respectively, whereas less than 3 members comprised subfamilies UBC1, UBC11, and UBC12. The change in the number of ZmUBC members suggested that the ZmUBC family had undergone lineage-specific expansion and functional divergence during the course of evolution. Phylogenetic data also suggested that UBC9 and UBC15 expanded in monocots but not in *Arabidopsis*. In subclasses UBC9 and UBC15, one and four members were detected in *Arabidopsis*, whereas eight and three members were present in maize and rice genome, respectively, indicating that UBC-encoding genes of these subclasses expanded in a species-specific manner from common ancestral genes that were present prior to the diversification of the monocot and dicot lineages. Relationship analysis of intragroup members with well-supported bootstrap values revealed that all of the 13 subfamilies included both proteins from eudicots and monocots, which indicated that the defined subfamilies were already present in the common ancestor of both groups, and evolved prior to the divergence of monocots and dicots. *Arabidopsis* and maize have at least one close homolog to the 15 UBC consensus enzymes of yeast. However, there are three UBC gene families (UBC14,UBC15,UBC16) in *Arabidopsis* and maize for which no probable orthologs exist in the budding yeast, whereas all three groups have potential orthologs in animals [64], which was indicative of possible gene loss during yeast evolution.

Previous reports have shown that plant E2 genes are involved in developmental and physiological processes, as well as biotic/abiotic stress responses under normal and stressed growth conditions [23]. Therefore, these are important factors for various plants to withstand adverse environmental conditions. The expression of UBC E2 genes in a number of plant species are regulated by tissues and/or developmental stage, as well as by environmental conditions [23, 27, 65]. In a previous report, three rice genes (*OsUBC2*/*5*/*18*) and five *Arabidopsis* genes (*AtUBC13*/*17*/*20*/*26*/*31*) in the UBC family were significantly downregulated, whereas only three rice genes (*OsUBC13*/*15*/*45*) were significantly upregulated under salt and drought stresses [55]. In maize, however, *ZmUBCs* showed different expression profiles in *Arabidopsis* and rice. Our data showed that over half of the *ZmUBC* genes (48 genes) and 16 *ZmUBC* genes were significantly upregulated under salt and drought condition, respectively. In *Arabidopsis*, the expression of *AtUBC24* (*PHO2*) and *AtUBC32* was upregulated by salt stress, and their mutants showed a reduction in the uptake and accumulation of Na^+^, leading to enhanced salt tolerance [27, 51]. Our data showed that *ZmUBC56*, *ZmUBC60*, and *ZmUBC71*, orthologs of *AtUBC24* and *AtUBC32*, respectively, had high expression levels under salt stress. Similar expression patterns suggested that these genes might play important roles under salt-stressed conditions during maize development. Contrary to *ZmUBC71*, another orthologous gene of *AtUBC32*, *ZmUBC62*, was highly expressed under cold stress. The observed differences in expression profiles between duplicated gene pairs, *ZmUBC62* and *ZmUBC71*, indicated that these genes might have undergone significant diversification after the duplication of the respective genomic segments, leading to neo-functionalization of the paired partners. These data indicated that *ZmUBCs* might play different roles under abiotic stresses compared to rice and *Arabidopsis*.

## Conclusions

It is essential to systematically analyze the members of the ubiquitin-conjugating enzyme family to elucidate its functions in plant development and stress response. In the present study, we present the genome-wide identification and analysis of the ubiquitin-conjugating enzyme families in maize. A total of 75 *ZmUBC* genes were identified in the maize genome. Phylogenetic analysis of maize, *Arabidopsis*, and yeast indicated that these ubiquitin-conjugating enzymes could be divided into 13 E2 and 2 UEV groups. Comparison of the number of UBC-encoding genes and mRNAs within and between species indicated that both gene duplication and alternative splicing contributed to the genomic complexity and proteomic diversity of *ZmUBC* genes. Analysis of exon-intron junctions and the conserved motif of each candidate gene has revealed high levels of conservation within and between phylogenetic groups. Clear orthologous relationships were established for the majority of UBC-encoding genes, facilitating in the generation of functional inference of the maize ubiquitin-conjugating enzymes. Although nearly all the *ZmUBC* genes were expressed in the examined organs, some genes were upregulated in one or several specific organs. The expression of some E2s could be up- or downregulated by different abiotic stress treatments, indicating the critical roles of this gene family in maintaining maize normal growth under stress conditions. However, additional studies on the detailed functions of each gene are warranted.

## Supporting Information

S1 Fig Phylogenetic tree (NJ tree) of ZmE2s. The NJ tree includes 75 UBC proteins from *Zea mays*, 15 from *Saccharomyces cerevisiae*, and 48 from *Arabidopsis thaliana*. The tree shows 15 phylogenetic subgroups depicted in various colors to distinguish diversification of subfamilies into clusters.
(TIF)

S2 Fig Differential expression levels of ZmUBC genes which increased or decreased by >15-fold in NaCl treatment, or increased or decreased by >15-fold in PEG 6000 or 4°C treatment. The X-axis indicates time course/treatment and the Y-axes are scales of relative expression levels. The maize *actin* gene was used as internal control. The presented data are representative of three independent experiments.
(TIF)

S1 Table Primers used in RT-qPCR of *ZmUBC* genes. (DOC)
