# Supplementary figures and images for: Genome-Wide Identification, Phylogenetic and Expression Analyses of the Ubiquitin-Conjugating Enzyme Gene Family in Maize
Source: PLoS One. 2015 Nov 25;10(11):e0143488. doi: 10.1371/journal.pone.0143488 (PMC4659669; doi:10.1371/journal.pone.0143488)

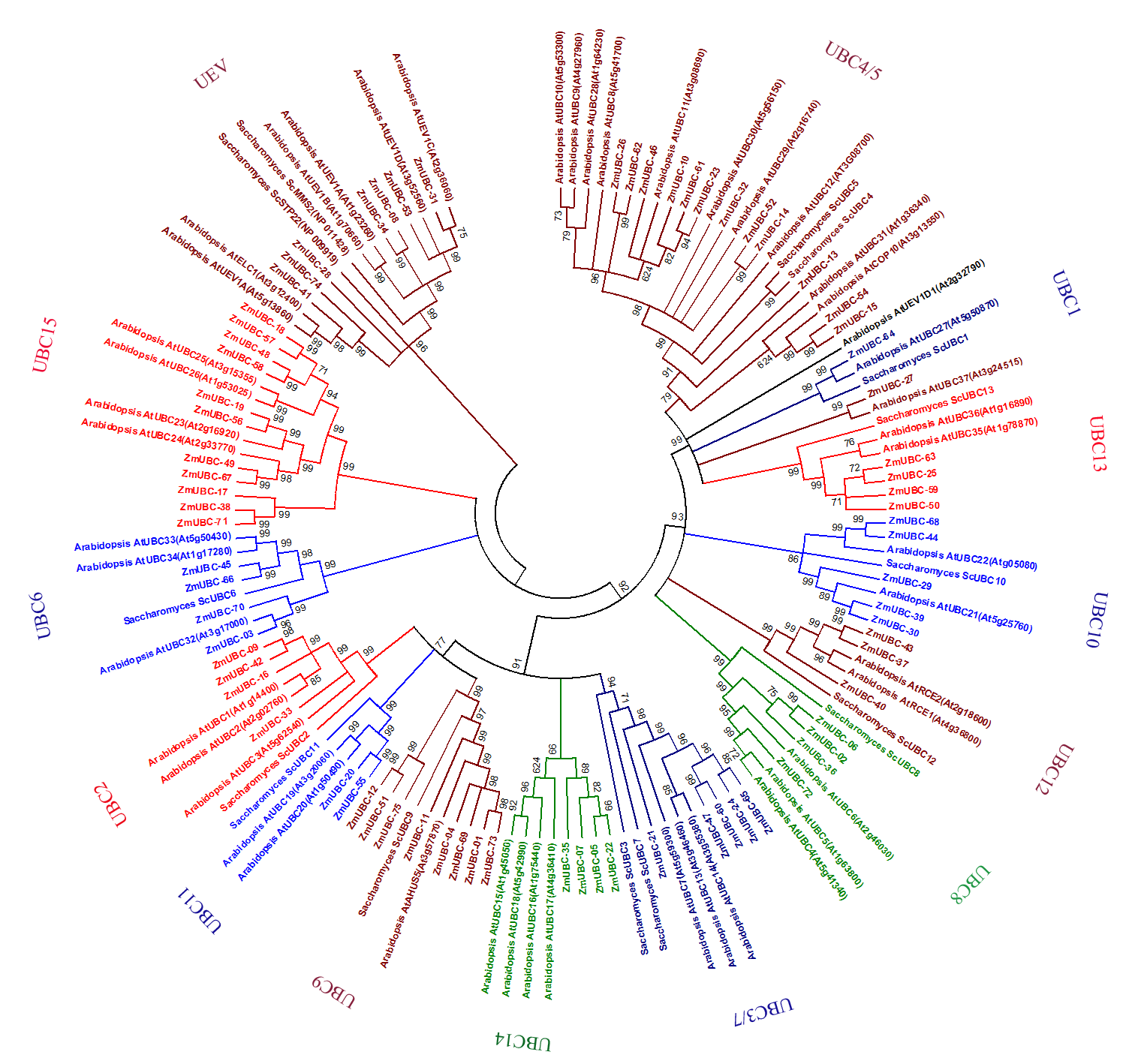

Supplement: S1 Fig — The NJ tree includes 75 UBC proteins from Zea mays, 15 from Saccharomyces cerevisiae, and 48 from Arabidopsis thaliana. The tree shows 15 phylogenetic subgroups depicted in various colors to distinguish diversification of subfamilies into clusters. (TIF) [file pone.0143488.s001.tif]

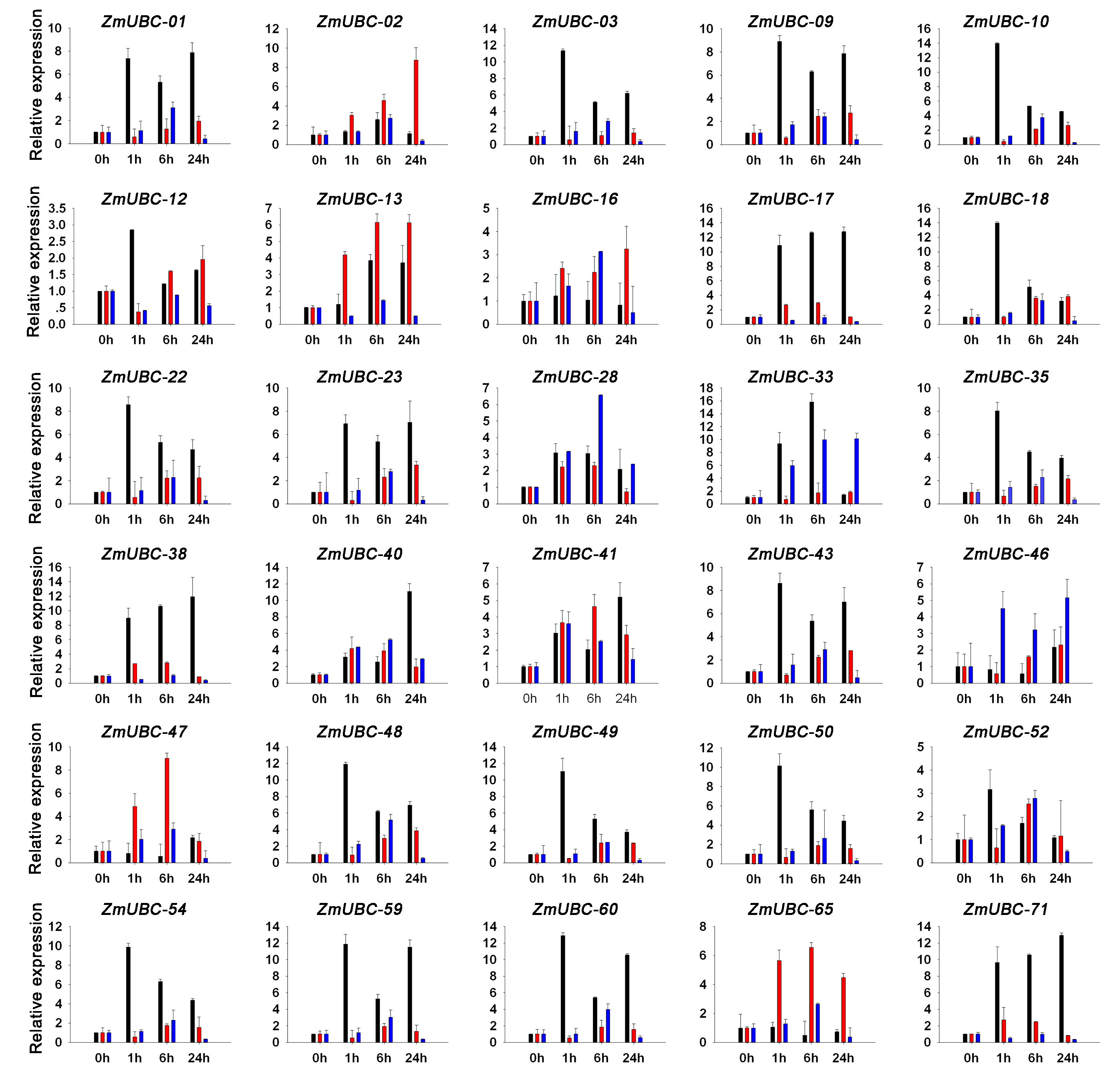

Supplement: S2 Fig — The X-axis indicates time course/treatment and the Y-axes are scales of relative expression levels. The maize actin gene was used as internal control. The presented data are representative of three independent experiments. (TIF) [file pone.0143488.s002.tif]
