# Supplementary material for: Genome-Wide Identification, Phylogenetic and Expression Analyses of the Ubiquitin-Conjugating Enzyme Gene Family in Maize
Source: PLoS One. 2015 Nov 25;10(11):e0143488. doi: 10.1371/journal.pone.0143488 (PMC4659669; doi:10.1371/journal.pone.0143488)
Supplement: S1 Table — (DOC) [file pone.0143488.s003.doc]

**S1 Table. Primers used in RT-qPCR of *ZmUBC* genes.**

| **Genes** | **Primers used in RT-qPCR(5′→3′)** | |
| --- | --- | --- |
| *ZmUBC-01* | Forwards： | AGCAAGCCTCCCAAGTGCAA |
|  | Reverse： | TGTTTGAGCAGGGTCAGCAGGA |
| *ZmUBC-02* | Forwards： | GCGTATCCTTACAAATCCCCATCGA |
|  | Reverse： | CACGCATCATCAACGCAGCT |
| *ZmUBC-03* | Forwards： | CCAAATGTGCCAAACGAGGA |
|  | Reverse： | TCTCTCTGTGGCTTGCTTCA |
| *ZmUBC-04* | Forwards： | GAGCTGTACCATCCCCGGCAA |
|  | Reverse： | CAGTGATAGCAGGTCTCCAACCACT |
| *ZmUBC-05* | Forwards： | TGCAGGTTGACTTCCCTGAGCA |
|  | Reverse： | CGGCAGTTCCTAACATAGCGGTCA |
| *ZmUBC-06* | Forwards： | TGCTGTTCTTGTTTCCCCGCTCT |
|  | Reverse： | CCGATGGACGGGGATTTGTAAGGA |
| *ZmUBC-07* | Forwards： | CCGATGGACGGGGATTTGTAAGGA |
|  | Reverse： | CTTCAAGCACAGGGTCACCGACGA |
| *ZmUBC-08* | Forwards： | GCATTGGTGATGGAACTGTG |
|  | Reverse： | TCCCACTTTCCAAGCATAGG |
| *ZmUBC-09* | Forwards： | ATTACCCCAACAAACCTCCAACTGT |
|  | Reverse： | CTGCTCCACAACCTCACGAACT |
| *ZmUBC-10* | Forwards： | ATTCACTTCCCACCGGATTACCCTT |
|  | Reverse： | GAGCGTGCGGTGGACTCATACTT |
| *ZmUBC-11* | Forwards： | CTCCCCACTGCAAGTTTCCAGCA |
|  | Reverse： | CGCTGGGGAGGCTGGATTT |
| *ZmUBC-12* | Forwards： | CCAAGTAACCCCCCAACCTGCAA |
|  | Reverse： | CTACACGAGCGACGGATACCTCTT |
| *ZmUBC-13* | Forwards： | GCAGCAAGGTTCTGTCATGCCTA |
|  | Reverse： | TCACCAAGAATACCCCACCTGCA |
| *ZmUBC-14* | Forwards： | GGATCTGCAAAAGGACCCACCGA |
|  | Reverse： | AGATGCTCCCGTTAGAGTTGATGT |
| *ZmUBC-15* | Forwards： | AAGGATCGCCATATGAACGAGGGAT |
|  | Reverse： | CAAGCGGGGTGTATGGATCTGGA |
| *ZmUBC-16* | Forwards： | CCCCGCACAGTCACCCTATGA |
|  | Reverse： | CTGGATTTGGGGCACTCAGAAGT |
| *ZmUBC-17* | Forwards： | GGCAAGGGGTGCGAGAAGT |
|  | Reverse： | CCCAGCAGACGACCCAACGA |
| *ZmUBC-18* | Forwards： | ATTCCCTCTGCTTATCCTTGTGCT |
|  | Reverse： | GCATAGGACTCATATCCTGGCTCGT |
| *ZmUBC-19* | Forwards： | CGTACCTGCTGAGCGTGAAATCCA |
|  | Reverse： | GGTCGCAGTCAGCTCCAGTGT |
| *ZmUBC-20* | Forwards： | ATCATGCTCTGGAACGCCGTCAT |
|  | Reverse： | TTGGATCACACAGCAGGGACTGA |
| *ZmUBC-21* | Forwards： | CCTGCTTCCACCCCAACGTCGA |
|  | Reverse： | CGCGGCGGGCTTGTAGATTTTCT |
| *ZmUBC-22* | Forwards： | ACCCGCCATCAGTCAGATTTACCT |
|  | Reverse： | CCTCAAGTTCCATTGGCTGCTT |
| *ZmUBC-23* | Forwards： | CCAAACAGCCCGCCATCAGT |
|  | Reverse： | TCCTCCCTCTTATCCCTCCATTCCT |
| *ZmUBC-24* | Forwards： | GACCTACCAGTTGCAGGTTGACTT |
|  | Reverse： | GCTTTGCTGGCGAACTAGACAACA |
| *ZmUBC-25* | Forwards： | GCAAAAGGACCCTCCAACGTCAT |
|  | Reverse： | GGATCGTCTGGGTTGGGATCAGT |
| *ZmUBC-26* | Forwards： | TCCTTTCAAACCACCAAAGG |
|  | Reverse： | GGACTCCACTGGTCCTTCAA |
| *ZmUBC-27* | Forwards： | CTCAAGGACAAATGGAGCCCAGCA |
|  | Reverse： | CGAGTCCACTCCTTGGCTGT |
| *ZmUBC-28* | Forwards： | GGTACCCTTTTCAACCACCGAATGT |
|  | Reverse： | CATCATCCGGGTTTGGCTCACT |
| *ZmUBC-29* | Forwards： | CAGTATCCTCTGCTGCCTCC |
|  | Reverse： | AGTGGGTTGTCTGGTTCAGG |
| *ZmUBC-30* | Forwards： | AGCACTGCCCTTCTTCCAACGA |
|  | Reverse： | AGCCCTCGCATCAGCTCCAT |
| *ZmUBC-31* | Forwards： | TCCAAGGAAGTTTGGTTTGC |
|  | Reverse： | GGAGGCTGCACTAACTTTCG |
| *ZmUBC-32* | Forwards： | CTCTGCTGCCTCCTCAAGTTCGA |
|  | Reverse： | ACAGTTAAGTGGGCTGTCTGGTT |
| *ZmUBC-33* | Forwards： | CCCAAGGTGGCGTTCAAGACGAA |
|  | Reverse： | ACTTGTGCGTCCATCCCCT |
| *ZmUBC-34* | Forwards： | GCATTGGTGATGGAACTGTG |
|  | Reverse： | GGTAGATTCGTCCCTCGTGA |
| *ZmUBC-35* | Forwards： | ATTACCCCAACAAACCTCCGACT |
|  | Reverse： | GCTCTGCTCCACAACCTCACGA |
| *ZmUBC-36* | Forwards： | GGGCACCAGGCACGCTCTACA |
|  | Reverse： | ATTGCTGGGGACCACGAGTCA |
| *ZmUBC-37* | Forwards： | TCGATGAGATGTCTGGCTCTGTCT |
|  | Reverse： | TGCCAGCGTCCTCTGGTTTAGCAT |
| *ZmUBC-38* | Forwards： | CTCGGCTGAAACGACAGGTGGTGA |
|  | Reverse： | TGTAGAGCGTCAGCGACTTGTGGA |
| *ZmUBC-39* | Forwards： | CCTCATGATCCTCCGAAGGTCA |
|  | Reverse： | CTCCGGTCATGGCTCTTTTCACAT |
| *ZmUBC-40* | Forwards： | CCTCTACTGCCTCCTCAAGTTCGA |
|  | Reverse： | TAAGTGGGCTGTCTGGTTCAGGAT |
| *ZmUBC-41* | Forwards： | CAGCCAGAATCACACGACAC |
|  | Reverse： | ATGAGCCACTTGACGTCCTC |
| *ZmUBC-42* | Forwards： | GACGTGTGGCAAGTGGAGGCGAA |
|  | Reverse： | ACCCATGTAGTATCCTTCATCAGGCT |
| *ZmUBC-43* | Forwards： | CGCCCCGCAGGATAACAACA |
|  | Reverse： | GCTCCACGACCTCCCGAACT |
| *ZmUBC-44* | Forwards： | CTCAACCTGCCGAAGACCACATCA |
|  | Reverse： | CGCGCAGAATGTTCAGACAGACA |
| *ZmUBC-45* | Forwards： | CACTGCCCAACGACATATTG |
|  | Reverse： | AGGACAGAAGCCCAGTGAGA |
| *ZmUBC-46* | Forwards： | CCCTCAATCTCCCCCAAAAGGAT |
|  | Reverse： | CGAGTAGCATCTTCCCAGCTTGT |
| *ZmUBC-47* | Forwards： | TCCACCGGACTACCCATTCAAACCA |
|  | Reverse： | GGACCAACGGATCGTCAGGGTT |
| *ZmUBC-48* | Forwards： | TACTCCTTACCATGACGGGCTCT |
|  | Reverse： | GCCGGGTTCCACTTCTCACAT |
| *ZmUBC-49* | Forwards： | GGACAGGCACTGGTAACGAAGTGT |
|  | Reverse： | GGCAGGTGAAATGCTCCTTGACCA |
| *ZmUBC-50* | Forwards： | GGACCGCCTGACACCCTATATGAT |
|  | Reverse： | CTCCAATCCTTGGCAGCTTCGAT |
| *ZmUBC-51* | Forwards： | CGCCCAAGAAAACCATTGCCACA |
|  | Reverse： | ACAGGCATACATCCCCAGAATCA |
| *ZmUBC-52* | Forwards： | CCTCCTGACTACCCCTTTAAGCCT |
|  | Reverse： | GTCGGTGAGGAGCGAGCTGAT |
| *ZmUBC-53* | Forwards： | GCTTGAACGTGGAGAGAAGG |
|  | Reverse： | GATGGTGGCTTCTCAGGGTA |
| *ZmUBC-54* | Forwards： | AAGGGCGACAACCTCTACCACT |
|  | Reverse： | AAGCGGGGAGTATGGATCTGGA |
| *ZmUBC-55* | Forwards： | CGGCAACATCTGCCTGGACAT |
|  | Reverse： | GGCGGCCTTGTAGAGCTTCTCCA |
| *ZmUBC-56* | Forwards： | CTGTTGAAGGGGAGAAGAATGCAGT |
|  | Reverse： | AGAGTGCCAACAACATTTCCTTGCA |
| *ZmUBC-57* | Forwards： | TATCCTTCTGGCCCTCCGTTGGT |
|  | Reverse： | GGAGTGGTGGCGAAGGACTCATAT |
| *ZmUBC-58* | Forwards： | GACTCCTTACCATGACGGGCTCT |
|  | Reverse： | TTGGCCGGATTCCACTTCTCACA |
| *ZmUBC-59* | Forwards： | AGCTTGGTAGGATATGCCTCGACA |
|  | Reverse： | CGAGTCCACTCCTTCGCTGTCT |
| *ZmUBC-60* | Forwards： | CCCAACAGCCCGCCATCAGTAA |
|  | Reverse： | TGCTGGAGACTCGTCATTCGGACT |
| *ZmUBC-61* | Forwards： | GCAAGCAACAATTATGGGACCACCT |
|  | Reverse： | GAGCAGATGGAGAGCAGGACCTT |
| *ZmUBC-62* | Forwards： | CTGATAGCCCATATTCTGGTGGAGT |
|  | Reverse： | TCGTCGGGGTTCGGATCGGTCA |
| *ZmUBC-63* | Forwards： | GCTGCCCCAAAGGTTAGGTTTCT |
|  | Reverse： | CGTCTGGATTTGGCGCACTCAGT |
| *ZmUBC-64* | Forwards： | CGCTCCTTTCCCTTCAAGCTCT |
|  | Reverse： | TCGTCGCCATCCACGCTCT |
| *ZmUBC-65* | Forwards： | CAAACAGCCCGCCATCAGTCAGA |
|  | Reverse： | TGCCTCAATGTTTGCTGGAGACTCA |
| *ZmUBC-66* | Forwards： | TGGAAGCATCAAGACGTCAG |
|  | Reverse： | GATGGAACAGGTGCAGGATT |
| *ZmUBC-67* | Forwards： | GACAGGCACTGGTAACGAAGTGT |
|  | Reverse： | AATGCTCCTTGACCAACGTCTCA |
| *ZmUBC-68* | Forwards： | TGTGAGATGCCTTCTGATTGAACCA |
|  | Reverse： | GGGGTTCTGATCCTGCAAACTCGT |
| *ZmUBC-69* | Forwards： | GCAAGAATCACCCCCACGGTT |
|  | Reverse： | AGGTCTCCAATCGCTATCCTCGT |
| *ZmUBC-70* | Forwards： | AGGACATCTTCGAGTGGCAA |
|  | Reverse： | TTCACCCGTGTTTTCTGCAG |
| *ZmUBC-71* | Forwards： | ACGGCCTTCGTCAGATCATGCAA |
|  | Reverse： | GTTTCCAGCTCCATCACTCGTCT |
| *ZmUBC-72* | Forwards： | GGTGGAGATGGTGAACGATGGGAT |
|  | Reverse： | GATCACGCATCATCAACGCAGCT |
| *ZmUBC-73* | Forwards： | CCTAGCAAGCCTCCCAAGTGCA |
|  | Reverse： | GACCAACGCGGGATACTGCTT |
| *ZmUBC-74* | Forwards： | CTGTCCTGGCTGCACAAGA |
|  | Reverse： | ACGCTTGATGTACTCGTGGA |
| *ZmUBC-75* | Forwards： | GGGGAAAGGGTAAACCAAAA |
|  | Reverse： | GGCAGCTGCGTAATAAAAGC |
| *ZmActin* | Forwards： | TCACTACGACTGCCGAGCGAG |
|  | Reverse： | GAGCCACCACTGAGGACAACATTAC |
